# Effect of Cocoa Bean Shell Addition on Metabolite Profile and Antioxidant Activity of Herbal Infusions

**DOI:** 10.1155/2021/9915797

**Published:** 2021-05-06

**Authors:** Maria Quijano-Avilés, Ivan Chóez-Guaranda, Rafael Viteri, Ana Barragán-Lucas, Daynet Sosa, Patricia Manzano

**Affiliations:** ^1^Centro de Investigaciones Biotecnológicas del Ecuador (CIBE), ESPOL Polytechnic University, Escuela Superior Politécnica del Litoral, ESPOL, Campus Gustavo Galindo Km 30.5 vía Perimetral, P.O. Box 09-01-5863, Guayaquil, Ecuador; ^2^Facultad de Ciencias de la Vida (FCV), ESPOL Polytechnic University, Escuela Superior Politécnica del Litoral, ESPOL, Campus Gustavo Galindo, Km. 30.5 vía Perimetral, P.O. Box 09-01-5863, Guayaquil, Ecuador

## Abstract

Cocoa bean shell (CBS) is a by-product with aromatic characteristics that can enhance the aroma and bioactivity of herbal infusions. This study was aimed to determine the effect of the addition of cocoa bean shell on the metabolite profile and antioxidant activity of infusions made with *Ilex guayusa* and *Vernonanthura patens* and their mixtures. Metabolite profile was analyzed by gas chromatography–mass spectrometry combined with multivariate analysis. Total polyphenol content and flavonoids were determined by the Folin-Ciocalteu method and by the flavonoid-AlCl3 complex, respectively. Antioxidant activities were measured by the decolorization assay of the 2,2-diphenyl-1-picrylhydrazyl radical and the ferric reducing antioxidant power. The results revealed that the addition of CBS increases the content of phenolic acids in the infusions (caffeic acid, 4-hydroxybenzoic acid, and pyrocatechol). Nonetheless, the antioxidant activity of the infusions decreased with the addition of CBS (16.21 to 2.74 TEAC). Carboxylic acids and derivatives, major compounds present in the infusions prepared with *V. patens*, were the metabolites that showed the highest correlation with the antioxidant activity. This study suggests that the infusions made with CBS present a profile of metabolites different from the infusions of *I. guayusa*, *V. patens*, and their mixtures.

## 1. Introduction

Herbal infusion is a widely consumed beverage and represents an important source of polyphenols [1]. Those bioactive compounds have been related to biological activities such as antioxidant, anti-inflammatory, and anticarcinogenic [2–4].

*Ilex guayusa* is a holly species that is consumed as a stimulant beverage attributable to the high caffeine content [5]. Although there is limited information about the biological properties of *I. guayusa*, recent studies have reported the species as antioxidant and anti-inflammatory because of the presence of polyphenols, flavonoids, xanthines, and carotenoids [6–11].

*Vernonanthura patens* is a wild plant distributed from Mexico to Argentina [12]. Leaves are used to prepare decoctions to calm headaches and for treating certain types of cancer [13]. Several compounds have been identified in the species such as pentacyclic triterpenoids, polyphenols, tannins, and flavonoids [14–16].

Cocoa bean shell (CBS) is a by-product of the cocoa industry [17]. The estimated generation of this material is about 700 thousand tons [18, 19]. Due to its nutritional characteristics such as fiber content, polyphenols, and lipid profile, many authors have focused on the use of this material to create value-added products [20–25]. For instance, Kraft Food has developed a patent to use CBS as a food additive that can improve the viscosity of dairy products and accentuate the chocolate flavour [26].

Therefore, the present study was aimed to establish the metabolite profile of herbal infusions prepare with *I. guayusa*, *V. patens* with adding CBS. Further, changes in antioxidant activity were also investigated and related with the metabolites identified using Pearson's correlation coefficient.

## 2. Materials and Methods

### 2.1. Reagents and Chemicals

Folin–Ciocalteu's phenol reagent 2 N, 2,2-Diphenyl-1-picrylhydrazyl (DPPH), 2,4,6–Tris (2-pyridyl)-s-triazine (TPTZ), 6-hydroxy-2,5,7,8-tetramethylchroman-2-carboxylic acid (Trolox), aluminium chloride hexahydrate (AlCl_3_), N,O-Bis(trimethylsilyl)trifluoroacetamide (BSTFA), quercetin, gallic acid, sodium nitrite, and methanol were acquired from Sigma–Aldrich (St. Louis, MO, USA). Saturated alkanes standard (C7-C40) was purchased from Supelco (Bellefonte, PA, USA). Sodium hydroxide, hydrochloric acid, and ethanol were obtained from J.T. Baker (Phillipsburg, NJ, USA). Sodium carbonate (Na_2_CO_3_) was purchased from Fisher Scientific (Lisbon, Portugal), acetic acid was from Panreac (Barcelona, Spain), and ferric chloride (FeCl_3_) was from Mallinckrodt (New York, NY, USA). Water was purified in a Milli-Q water purification system Millipore (Bedford, MA, USA).

### 2.2. Preparation of Herbal Infusions

An augmented simplex-centroid mixture design with three components and ten formulations was employed in order to evaluate the addition of CBS in the infusions. According to Table 1, different proportions of CBS, *I. guayusa*, and *V. patens* were used. CBS were provided by Maquita Cushunchic-MCCH (nonprofit foundation), Guayaquil, Guayas. *I. guayusa* (voucher No. CIBE020), and *V. patens* (voucher No. CIBE037) leaves were obtained from Taisha, Morona Santiago, and Marcabelí, El Oro, respectively. Samples of plant material were authenticated by the National Herbarium of Ecuador. Then, the remaining plant material was dried, ground, and sieved individually. Afterward, herbal formulations were prepared by pouring 200 mL of boiled distilled water over 2 g of raw material (CBS, *I. guayusa* and *V. patens*) for 5 min without mixing. Infusions preparation were filtered through a paper Whatman #1 and kept at -17°C until its use.

### 2.3. Metabolite Profile by GC-MS

Metabolite profile was performed by gas chromatography–mass spectrometry (CG–MS) (Agilent Technologies 7890A GC system and 5975C inert XL MSD with a triple axis detector), using a DB-5MS capillary column (30 m length × 0.25 mm i.d.×0.25 *μ*m film thickness, Agilent Technologies, Inc.) and helium as a carrier gas at a flow rate of 0.6 mL/min [27]. Briefly, 5 mg of freeze-dried infusion was mixed with 200 *μ*l of BSTFA, and incubated in a water bath at 70°C for 2 hours. After derivatization, 2 *μ*l of samples were injected at 280°C with splitless mode, and three biological replicates of each sample were measured. The initial oven temperature was held at 70°C for 5 minutes, after it was increased to 130°C at 15°C/min, then it was increased to 160°C at 4°C/min (held for 15 minutes), and finally, it was increased to 300°C at 10°C/min (held for 15 minutes). The MSD transfer line was 285°C, and the ion source temperature was 230°C. Electron ionization of 70 eV was used, and the data compounds were collected with the full scan mode (40-700 amu) in the quadrupole mass analyzer.

### 2.4. GC–MS Data Processing and Compound Identification

Raw data files were converted to NetCDF/AIA (^∗^.cdf) format using the ChemStation GC/MSD Data Analysis Software (Agilent Technologies, Palo Alto, CA, USA). MzMine 2 (version 2.29) was employed for mass spectra detection, chromatographic building, deconvolution, and alignment [28]. Next, the resulting data sets were imported into MetaboAnalyst 3.0 for multivariate statistical analysis [29]. Compounds were tentatively identified by matching mass spectra with the information available in the NIST 11 Wiley 9 database and by comparing the estimated retention index using a series of *n*-alkanes (C7-C40) [30, 31].

### 2.5. Antioxidant Capacity of Herbal Infusions

Total phenolic content (TPC) was determined spectrophotometrically using Folin–Ciocalteu method [32], and the results were expressed as mg Gallic Acid Equivalent (GAE)/L. Total flavonoid content (TFC) was estimated according to the aluminium chloride colorimetric method [33], and the results were expressed as mg Quercetin Equivalent (QE)/L. Scavenging activity of CBS infusion against DPPH free radical was calculated by referenced method [34], and results were expressed as an equivalent of mM Trolox (TEAC). Finally, the Ferric Reducing Antioxidant Power (FRAP) was evaluated by the reducing power of ferric-tripyridyl-triazine (Fe3+-TPTZ) complex to ferrous form (Fe2+) method [35]. Then, a standard curve of Trolox was calculated, and the results were expressed as equivalent of mM Trolox (TEAC).

### 2.6. Statistical Analysis

All experiments were conducted in triplicate and values were expressed as mean ± standard deviation (SD). Statistical significance was analyzed through ANOVA and Tukey test at *p* < 0.05 using the statistical software package Minitab 16 (Minitab Inc., State College, PA, USA).

## 3. Results and Discussion

### 3.1. Multivariate Analysis of Herbal Infusions Using Different Mixture of Raw Materials

**Figure 1:**
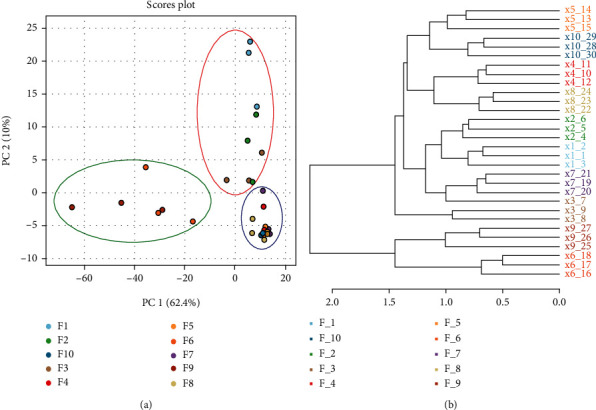
(a) Principal component analysis (PCA) of herbal infusions prepared with cocoa bean shell, *I. guayusa* and *V. patens* from the GC-MS data. (b) Dendrogram resulting from a hierarchal cluster analysis based on the distribution of all detected metabolites.

Metabolite profiles of ten formulations obtained by a mixture design of experiment were assessed by CG–MS in order to determine the effect of CBS addition on herbal infusions chemical composition of *I. guayusa* and *V. patens* medicinal plants. The principal component analysis (PCA) explained 72.40% of the total of variability (Figure 1(a)). The PC1 (62.40%) separated F6 and F9 from F1, F2, F3, F4, F5, F7, F8, and F10. The separation can be attributed to the quantity of CBS present in the infusions F6 and F9, which is higher than in the other formulations. However, F7 and F8 samples present an equal or higher content of CBS but they were not grouped by the presence of V. patens. Additionally, PC2 (10%) separated F1, F2, and F3 from F4, F5, F7, and F8, which indicated that I. guayusa and V. patens present a similar chemical profile, in contrast to CBS. Hierarchical cluster analysis (HCA) of all detected metabolites showed a similar pattern to that suggested by the PCA and divided the samples into two clusters at a distance of two in the dendrogram (Figure 1(b)).

### 3.2. Comparison of Metabolites in Herbal Infusions

**Figure 2:**
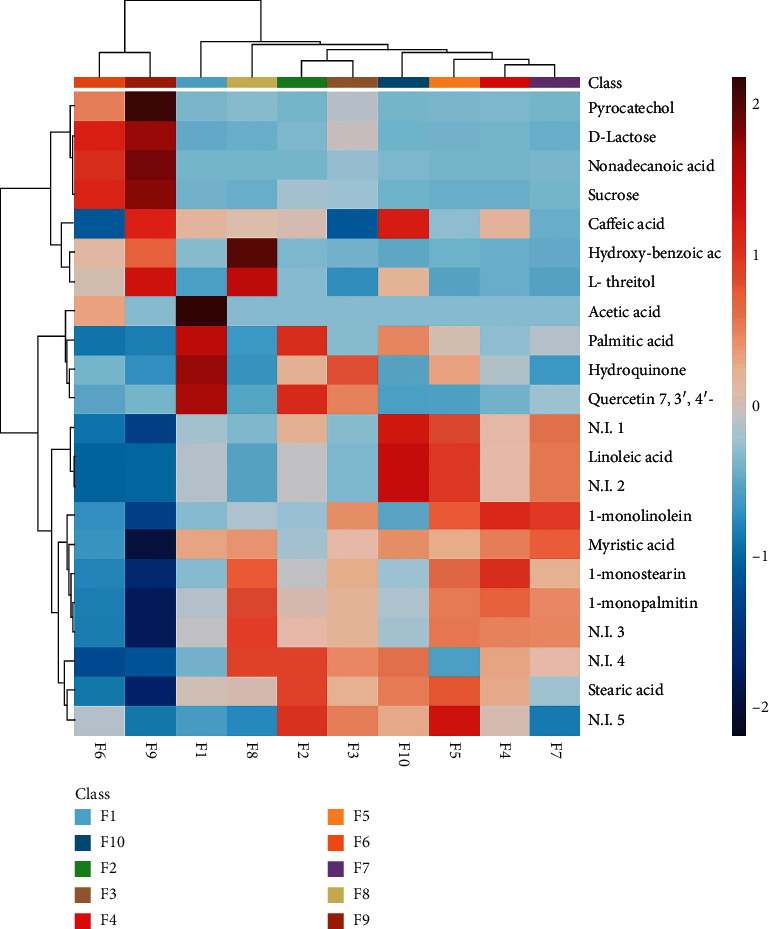
Heatmap representation of metabolite correlations in herbal infusions. Correlations coefficients were calculated based on Pearson's correlation.

Twenty-two metabolites were selected based on the variable importance in the projection (VIP) > 2 and *p* value < 0.05 in partial least squares discriminant analysis (PLS-DA). Carboxylic acids and derivatives (9), phenols (5), and sugar and sugar alcohols (3) were identified to discriminate the chemical composition of herbal infusions (Table 1). According to the heat map (Figure 2), carboxylic acids and derivatives were predominant in the formulations that contains *V. patens* as the main raw material (F3, F10, and F5). Among the phenolic acids, they were more abundant in the formulation (F9) that contains CBS (caffeic acid, hydroxy-benzoic acid, and pyrocatechol), followed by formulation (F2) with *I. guayusa* (quercetin 7,3′, 4′-trimethyl ether) and *V. patens* (F3) (hydroquinone). Sugar and sugar alcohols were found in the formulations with higher content of CBS (F9, F8, and F6).

### 3.3. Correlation between Bioactivity and Metabolite Composition of Herbal Infusions

**Figure 3:**
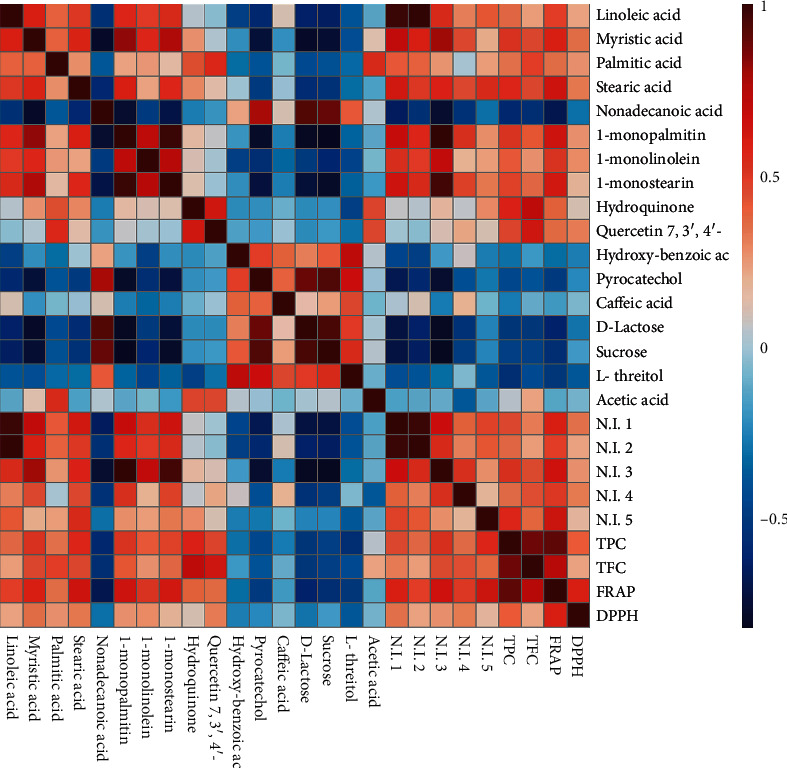
Correlation patterns among metabolites and antioxidant activity (DPPH, FRAP, TPC, and TFC). Each square indicates Pearson's correlation coefficient of a pair of metabolites and antioxidant activity. Red color represents positive correlation (0 < *r* < 1) and blue color represents negative correlation (−1 < *r* < 0).

Correlation map between antioxidant activity (DPPH, FRAP), TPC, TFC, and metabolite profile was performed in order to determine the potential compounds that are related to the biological activity of the infusions. According to Figure 3, fourteen compounds including linoleic acid, myristic acid, palmitic acid, stearic acid, 1-monopalmitin, 1-monolinolein, 1-monostearin, hydroquinone, quercetin 7,3′, 4′-trimethyl ether, and five nonidentified compounds showed a positive correlation with antioxidant activity, TPC, and TFC. On the other hand, eight compounds identified as nonadecanoic acid, hydroxy-benzoic acid, pyrocatechol, caffeic acid, d-lactose, sucrose, l-threitol, and acetic acid were negatively correlated to antioxidant activity, TPC, and TFC. Additionally, a strong positive correlation was observed between antioxidant activity and TPC and TFC.

### 3.4. Antioxidant Activity, TPC, and TFC of Herbal Infusions

**Figure 4:**
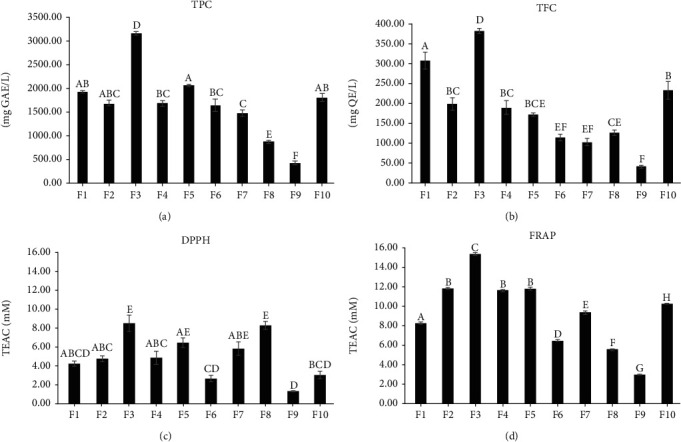
Antioxidant activity assay, total polyphenol content (a), total flavonoid content (b), DPPH (c), and FRAP (d) of herbal infusions prepared with cocoa bean shell, I. guayusa and V. patens. Values are expressed as mean ± SD. The same letter indicates values that are not significantly different by Tukey test at *p* < 0.05.

Antioxidant activity (DPPH, FRAP), TPC, and TFC of ten formulations of herbal infusions are showed in Figure 4. The antioxidant activity ranged from 8.74 (F3) to 1.41 (F1) mM TEAC for the DPPH assay and from 16.21 (F3) to 3.12 (F1) mM TEAC for FRAP assay. Higher values of TPC and TFC were observed in F3 (3306.04 mg GAE/L and 388.19 mg QE/L), and lower values were registered in F9 (428.18 mg GAE/L and 42.64 mg QE/L).

In this research, we studied how the addition of CBS in herbal infusion affects the chemical composition and antioxidant activity of beverages made with *I. guayusa* and *V. patens*. Despite, CBS was rich in phenolic compounds; the addition of this raw material only increased the content of caffeic acid and hydroxy-benzoic acid, which are compounds that have exhibited relevant biological activities. Caffeic acid has been related to the prevention of acute neuroinflammation-induced [36]. Moreover, analgesic and anti-inflammatory activities have been reported for hydroxy-benzoic acid [37]. On the other hand, infusions made with *I. guayusa* were characterized by the presence of quercetin 7, 3′, 4′-trimethyl ether, a methyl flavone that is reported for the first time to the species. Palmitic acid and stearic acid were also predominant in *I. guayusa* and have been reported in *I. paraguariensis* [38, 39]. Palmitic acid has been associated with the antimicrobial and antioxidant activities of *Scenedesmus intermedius* [40]. *V. patens* infusions presented high amounts of linoleic acid, myristic acid, hydroquinone, 1-monopalmitin, 1-monolinolein, and 1-monostearin. Unsaturated fatty acid, linoleic acid, has exhibited an inhibitory effect on AgRP expression suggesting that the compound can help to reduce food intake and treat obesity [41].

Antioxidant activity (DPPH, FRAP) was decreased with the addition of CBS. Nevertheless, F8 that presents an important content of CBS exhibited an antioxidant activity for the DPPH assay as high as the formulation elaborated using only *V. patens* (F3). According to previous study [42], DPPH activities reported for chamomile infusion (0.90 ± 0.02 mM TEAC) is lower than the values found for all the formulations of this study. Spearmint infusion (3.33 ± 0.11 mM TEAC) reports a higher antioxidant activity than F9 and F10 but lower than the other formulations. Black tea infusion presents a lower activity (5.13 ± 0.08 mM TEAC) than F3, F5, F7, and F8, and all ten formulations present a lower antioxidant activity than green tea infusion (24.62 ± 0.49 mM TEAC). In the case of FRAP activity, chamomile infusion presents a lower antioxidant activity (1.03 ± 0.14 mM TEAC) than the samples of this investigation. Only the formulation that consisted of 100% of CBS (F9) showed a lower FRAP value than spearmint (5.33 ± 0.09 mM TEAC) and black tea infusion (3.38 ± 0.01 mM TEAC). Green tea infusion has a higher FRAP value (24.98 ± 0.41 mM TEAC) than the formulations of this research.

CBS infusion (F9) presents a TPC higher than *Sideritis syriaca* (Greek Mountain tea infusion) [43], *Moringa oleifera* [44], and *Matricaria chamomilla* [45] infusions. However, TCP was lowered by the addition of CBS in *V. patens* and *I. guayusa* infusions. TPC of all formulations, with the exception of F9, are higher than the values reported for *Mentha piperita*, *Eucalyptus globules*, and *Salvia fruticose* but are lower than black and green tea [43]. In the case of TFC, the values described for the species V. patens, I. guayusa, and CBS are reported for the first time in the literature.

The compounds that mainly contribute to the antioxidant activity of the infusions are carboxylic acids and derivatives. Fatty acids have been reported as bioactive compounds in herbal medicines [46]. Linoleic, myristic, and palmitic acids have shown antioxidant activity by analysis of model liposome oxidation [47]. However, linoleic acid has not shown radical quenching activity against DPPH [48]. Additionally, in previous studies, stearic acid has not presented antioxidant activity [47]. In addition, phenolic compounds have also been reported as antioxidants [49]. Nevertheless, it was unexpected that caffeic acid did not show a positive correlation with antioxidant activity, because this compound has exhibited relevant biological activity such as antioxidant and anti-inflammatory [50].

## 4. Conclusions

This study showed that the addition of CBS to herbal infusions of *I. guayusa* and *V. patens* could increment the variety of polyphenols found in these raw materials. However, the addition of this by-product decreased the antioxidant activity of the infusions. *I. guayusa* and *V. patens* presented a metabolite profile clearly different from CBS, and antioxidant activity was correlated principally to the presence of carboxylic acids. These findings indicate that CBS is a good source of phenolic compounds.

## Figures and Tables

**Table 1:** Coded values of the herbal formulations prepared with cocoa bean shell (CBS), *I. guayusa* and *V. patens*.

Formulation	CBS (%)	*I. Guayusa* (%)	*V. patens* (%)
F1	0,33	0,33	0,33
F2	0	1	0
F3	0	0	1
F4	0,167	0,667	0,167
F5	0	0,5	0,5
F6	0,5	0,5	0
F7	0,5	0	0,5
F8	0,667	0,167	0,167
F9	1	0	0
F10	0,167	0,167	0,667

## Data Availability

All data have been placed in the manuscript.
